# Complete mitochondrial genome of *Sargassum yezoense* (Sargassaceae, Phaeophyceae)

**DOI:** 10.1080/23802359.2018.1457993

**Published:** 2018-04-01

**Authors:** Kyeong Mi Kim, Ji Won Choi, Hwan Su Yoon, Hyeong Seok Jang, Ji Won Hong

**Affiliations:** a Department of Taxonomy and Systematics, National Marine Biodiversity Institute of Korea, Seocheon, Republic of Korea;; b Department of Biological Sciences, Sungkyunkwan University, Suwon, Republic of Korea

**Keywords:** Complete mitochondrial genome, *Sargassum yezoense*, Sargassaceae, Phaeophyceae

## Abstract

The mitochondrial genome of *Sargassum yezoense* (Yamada) Yoshida & T. Konno from Uljin, Korea was completely sequenced. This mitochondrial genome has 34,787 bp in length and consists of 65 genes including 37 protein-coding, three rRNA, and 25 tRNA genes. The overall GC content of the genome is 36.5%.

*Sargassum* C. Agardh (Sargassaceae, Phaeophyceae) is a widely distributed genus on rocky intertidal shores worldwide and represents one of the most species-rich genera of the marine macrophytes (Mattio and Payri 2011). *Sargassum yezoense* (Yamada) Yoshida & T. Konno is a large fucacean brown alga that is distributed in Japan (Agatsuma et al. 2002) and Korea (Oak and Lee 2006). This alga forms forests or meadows in sublittoral regions serving as nursery habitats and spawning grounds for marine invertebrates and fish (Fuse 1962). In this investigation, the complete mitogenome of *S. yezoense* was determined for the first time. This new information would contribute to the preservation of its genetic resources and provide better understanding of phylogenetic relationships of the *Sargassum* species and mitochondrial genome diversity in the Fucales.

We collected *S. yezoense* from Uljin, Gyeongsangbuk-do, Korea (36° 59′ 24.41″N 129° 24′ 56.62″E) on 5 April 2017. The specimen was deposited at the National Marine Biodiversity Institute of Korea (MABIK) under the accession number AL00070893. The total genomic DNA was extracted from fresh sample using an Exgene Plant SV Mini kit (GeneAll Biotechnology, Seoul, Korea) followed by preparation of a library based on the manufacturer’s instruction (Ion Torrent, Guilford, CT). Total genomic DNA was sequenced using the Ion Torrent Personal Genome Machine (Life Technologies, Carlsbad, CA), and high quality data, ca. 1.75 Gb were obtained. Total reads were assembled using the MIRA V. 4.0.2.1 and the CLC *de novo* assembler included in CLC Genomics Workbench V.5.5.1 (CLC Bio, Aarhus, Denmark). The size of the circular mitogenome produced is 34,767 bp (GenBank accession number MG674825) which is similar to the previously reported *Sargassum* mitogenomes (34,606–34,925 bp). The nucleotide composition is 26.9% A, 36.6% T, 21.5% G, and 15.1% C. The overall GC content is 36.6%. The *S. yezoense* mitogenome contains 65 genes, including 37 protein-coding, three rRNA, and 25 tRNA genes. The ATG start codon is used in all protein-coding genes. Twenty eight protein-coding genes end with TAA stop codon, five genes with TGA, and four genes with TAG, respectively. It was found that there are 10 cases of gene-overlapping ranging from 1 to 16 bp in size. All 25 tRNA genes ranged from 72 to 88 bp in length showing the typical cloverleaf secondary structures.

Phylogenetic analysis was carried out using 11 reported brown algal mitogenome sequences (Liu et al. 2015; Liu and Pang 2015, 2016a, 2016b; Bi and Zhou 2016; Liu, Pang, Chen 2016; Liu, Pang, Luo 2016; Amaral-Zettler et al. 2017). *Sargassum yezoense* combined tightly with *S. muticum*, *S. hemiphyllum*, *S. thunbergii*, *S. fusiforme*, and *S. horneri* with strong support values (Figure 1). Based on the complete mitogenome of *S. yezoense*, new mt DNA marker could be selected and employed to study the phylogeography and phylogeny for Fucales.

**Figure 1.**
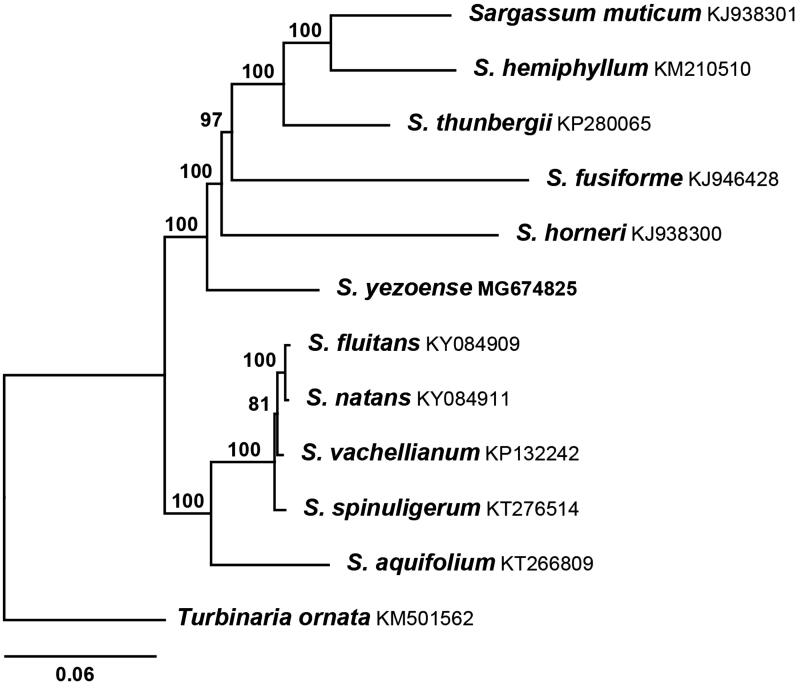
Maximum-likelihood phylogenetic tree of *S. yezoense* and 11 other species. GenBank accessions were indicated with species name except *S. yezoense*. The specimen of *S. yezoense* was deposited at the National Marine Biodiversity Institute of Korea (MABIK) under the accession number AL00070893.
